# FISH as an effective diagnostic tool for the management of challenging melanocytic lesions

**DOI:** 10.1186/1746-1596-6-76

**Published:** 2011-08-11

**Authors:** Mathew W Moore, Robert Gasparini

**Affiliations:** 1 NeoGenomics Laboratories. 12701 Commonwealth Drive, Suite 5, Fort Myers, FL 33913, USA

## Abstract

**Background:**

The accuracy of melanoma diagnosis continues to challenge the pathology community, even today with sophisticated histopathologic techniques. Melanocytic lesions exhibit significant morphological heterogeneity. While the majority of biopsies can be classified as benign (nevus) or malignant (melanoma) using well-established histopathologic criteria, there exists a cohort for which the prediction of clinical behaviour and invasive or metastatic potential is difficult if not impossible to ascertain on the basis of morphological features alone. Multiple studies have shown that there is significant disagreement between pathologists and even expert dermatopathologists in the diagnosis of this subgroup of difficult melanocytic lesions.

**Methods:**

A four probe FISH assay was utilized to analyse a cohort of 500 samples including 157 nevus, 176 dysplastic nevus and 167 melanoma specimens.

**Results:**

Review of the lesions determined the assay identified genetic abnormalities in a total of 83.8% of melanomas, and 1.9% of nevus without atypia, while genetic abnormalities were identified in 6.3%, 6.7%, and 10.3% of nevus identified with mild, moderate and severe atypia, respectively.

**Conclusions:**

Based on this study, inheritable genetic damage/instability identified by FISH testing is a hallmark of a progressive malignant process, and a valuable diagnostic tool for the identification of high risk lesions.

## Background

Malignant melanoma is the seventh most common cancer responsible for 1-2% of all cancer related deaths [1] with the fastest increase in incidence identified as 3-7% per year in the United States. Cutaneous malignant melanoma is a neoplasm which arises from melanocytes; it may occur de novo, or from a pre-existing lesion such as a congenital, acquired, or atypical (dysplastic) nevus. Noncutaneous primary sites of melanocytes include mucosal epithelium, retinas, and leptomeninges [2]. Melanoma is difficult to treat with therapy and most commonly fatal if not surgically removed prior to metastatic progression [3]. Early and accurate diagnosis of melanoma is therefore widely regarded as the most important factor in predicting outcome from this disease[4].

Successful management of potential melanomas is technically challenging. The initial lesion is usually discovered during either a routine physician visit, or dermatology check-up. The "ABCD" method of physical examination [5]**, A**symmetry, **B**order irregularity, **C**olor variegation, and a **D**iameter of 6 mm or more, in conjunction with a noticeable change over time, is typically utilized to determine the need for an initial biopsy.

Histopathologic analysis remains the gold standard for the pathologic diagnosis of melanoma. Numerous criteria for the histopathologic diagnosis of melanoma have been described including asymmetry, poor circumscription, cytologic atypia, mitotic activity, and failure of maturation with descent [6]. Even with these criteria, a considerable number of cases demonstrate conflicting morphologic criteria, and several studies have shown a level of intra-observer discordance at between 2-17% [6,7]. As Shoo et al. (2010) demonstrated, based on 1,500,000 biopsies, and a discordance of 14.3%, this equates to ~214,500 discordant cases in the USA annually.

### The Genetics of Melanoma

The melanocyte is a neural crest-derived cell that localizes in humans to several organs including the epidermis, eye, inner ear and leptomeninges [8]. In the skin, melanocytes synthesize and transfer melanin pigments to surrounding keratinocytes, leading to skin pigmentation and protection against solar exposure. The combination of a long life cycle, constant ultraviolet light exposure and other environmental stresses, and a high rate of cell division coupled to replicative senescence [9] categorize melanocytes as a high risk cell type for genetic mutation and selection. Progression from melanocyte through dysplasia, and melanoma has been shown to be an evolutionary process requiring multiple genetic events that subsequently affect several oncogene and tumor suppressor pathways, which ultimately result in increasing dysplasia, and progression to invasion, and metastatic potential [10]. This is consistent with current literature models in other cancer types outlining a progression from a normal cell type through dysplasia, and finally malignancy.

The characterization of melanocytic progression to melanoma presents specific challenges derived from the diverse phenotypic nature of the disease. While it is generally regarded that benign nevi contain few or no genetic alterations, and melanoma are known to possess frequent gross genetic alterations [7] there continues to be widespread discussion on the fundamental underlying biology of dysplastic nevi. Central to the argument is the contested proposition of the nature of melanocytic dysplasia; are a subset of dysplastic nevi premalignant lesions of melanoma, or are all dysplastic nevi fundamentally benign and genetically distinct from melanoma? Morphologically, and phenotypically, this is unclear as many studies have shown a wide disparity in diagnosis even amongst experts in challenging cases [11,12]. Current molecular studies provide convincing evidence defining the dysplastic nevi group as a heterogeneous population of normal chromosomal complement; defined point mutations, and less commonly, demonstrating a high level of chromosomal instability and imbalance [13-16]. In contrast to this heterogeneous nature, congenital nevi contain a normal chromosomal complement (although "initiating" mutations in BRAF and NRAS amongst others have been identified), while conversely, melanoma as a group exhibits a high degree of genetic instability with multiple gains, losses, and re-arrangements documented [14].

Recently, Gerami et. al. (2009) [7] described a Fluorescent *in situ *hybridization (FISH) based assay for distinguishing this "High Risk" group of nevi, based on copy number alterations of 3 genomic loci.. These regions contain the known cancer oncogenes CCND1 (11q23), MYB (6q22-q23) and RREB1 (6p25). Gerami et. al. (2009) demonstrated that these 3 loci were found predominantly in melanomas (87%), while rarely (5%) in congenital nevi. Significantly, a small percentage of dysplastic nevi (5%) were also shown to harbour this mutation. This study was able to identify a subset of atypical nevi which later progressed to melanoma, and through retrospective analysis, demonstrated that this group of nevi harboured one or more of the CCND1, P16 or RREB1 copy number alternations, effectively demonstrating that atypical nevi possessing these mutations have both invasive and metastatic potential.

Given the inherent difficulty in phenotypically distinguishing dysplastic nevi from melanoma, this paper aims to expand on the previous studies demonstrating that there exists a subset of melanocytic lesions for which a confident distinction of nevi from malignant melanoma cannot be made morphologically, but which can be resolved through FISH analysis.

## Methods

### Sample Set

The sample set for the validation study consisted of samples of known melanoma, atypical nevus and benign nevus (Table 1). All samples were obtained from three commercial reference laboratories, and had undergone a standard clinical diagnostic workup, including hematoxylin and eosin stain (H&E), and where required, special stains. Study sites were asked to provide as contiguous set of samples as possible (from each of the 3 groups) to limit selection bias. Samples were removed of all protected health information, and either blocks or slides were shipped to the study site. All samples were received with the original clinical diagnosis, which was retained by the study coordinator. Samples were subsequently re-examined and the original diagnosis reviewed by an internal board-certified dermatopathologist. A small number of cases for which a conflicting diagnosis was obtained were excluded from the validation study. Following the internal pathology review, all samples received an internal reference number, and the cases were blinded to the FISH technologists.

**Table 1:** Sample Set: Composition by Diagnosis.

	Total Cases	Positive By FISH	% Positive
**Nevus**			

Congenital	8	0	0.00%

Compound	37	0	0.00%

Intradermal	62	1	1.60%

Junctional	9	0	0.00%

Mild Atypia	32	2	6.30%

Blue	9	0	0.00%

		**Percent Positive**	**1.90%**

			

**Nevus-Dysplastic**			

Nevus-Moderate Atypia	60	4	6.70%

Nevus-Severe Atypia	29	3	10.30%

Nevus-Scalp	38	3	7.90%

Nevus-Spitz	49	6	12.20%

**Subtotal**	**176**	**Percent Positive**	**9.10%**

			

**Melanoma**			

Superficial Spreading	71	57	80.30%

Spindle cell/desmo-plastic	3	2	66.70%

Nodular	45	42	93.30%

In Situ	8	6	75.00%

Metastatic	40	33	82.50%

**Subtotal**	**167**	**Percent Positive**	**83.80%**

**Total Cases**	**500**		

### Study Design

This study followed the standard criteria for clinical validation of an analytical assay to the standards defined by the appropriate accrediting agencies. The following metrics were analyzed.

### Specificity

The assay specificity was determined from the benign nevus group. Receiver operator curve (ROC) analysis and plots of sensitivity versus specificity were used to independently document the relative power of each probe set at various FISH cutoffs. Final assay cutoffs were selected to maximize specificity over sensitivity.

### Sensitivity

Sensitivity was calculated as the percentage of unambiguous melanoma cases defined as positive by the 4-probe FISH assay using the derived FISH scoring cutoffs determined from the sensitivity experiment.

### Atypical Positivity Rate

The percentage of nevi with moderate or severe atypia that are scored positive using the FISH scoring cutoffs derived from the specificity experiment were determined.

#### FISH Hybridization

5 μm tissue sections were baked at 65°C for 20 min and deparaffinized through multiple xylene washes and dehydrated with ethanol. Specimen slides were air dried and subsequently passed through a series of pre-treatment washes on the VP2000 Processor (Abbott Molecular, Abbott Park, Illinois, U.S.A.): 1X SSC (80°C, 35 min), de-ionized (DI) water (3 min), protease solution (10% Pepsin in 0.2N HCL; 37°C for 34 min), DI water (3 min), increasing ethanol series (70%, 80%, 100% ethanol, 2 min each). The probe cocktail; MYB (6q23) in SpectrumGold; CCND1 (11q13) in SpectrumGreen; RREB1 (6p25) in SpectrumRed and D6Z1 (6 Centromere) in SpectrumAqua (Abbott Molecular, Abbott Park, Illinois, U.S.A.) was manually dispensed onto the specimen and covered with a cover glass. Denaturation was performed on the Thermobrite (Abbott Molecular, Abbott Park, Illinois, U.S.A) at 74°C for 8 min followed by the hybridization at 37°C for 12-16 hrs prior to coverslip removal. The post-hybridization wash procedure on the VP2000 consisted of 2X SSC with 3% Tween (70°C, 2 min) and 0.5% SSC prior to application of DAPI counterstain and signal enumeration.

### Enumeration of FISH Signals

Slides were analysed, digitally documented, and automated probe signal enumeration performed with a Metafer Slide Scanning System (MetaSystems, Waltham, MA). The algorithm of Gerami et. al. (2009) was used for scoring, with adjustments for cutoff values as described in the discussion. All areas for automated analysis were selected by a dermatopathologist. All analyses were performed blinded of the specimen's diagnoses. Tumor-bearing areas were localized using the DAPI filter at low magnification. The tumor area was then thoroughly inspected for the presence of nuclei harbouring abnormal copy numbers of any probe. Areas with the most significant copy number changes were selected for automated enumeration. Wherever possible, a minimum of 3 abnormal areas were selected and within each area 10 random nuclei were analyzed under high-power (64× oil objective). Overlapped or poorly hybridized nuclei were excluded from analysis. Samples in which less than 20 nuclei could be evaluated were excluded from the study.

## Results

Table 2 summarizes study cohort assay results.

**Table 2:** Study Results.

	Total Cases	Positive By FISH	% Positive
**Nevus**			

Congenital	8	0	0.00%

Compound	37	0	0.00%

Intradermal	62	1	1.60%

Junctional	9	0	0.00%

Mild Atypia	32	2	6.30%

Blue	9	0	0.00%

		**Percent Positive**	**1.90%**

			

**Nevus-Dysplastic**			

			

Nevus-Moderate Atypia	60	4	6.70%

Nevus-Severe Atypia	29	3	10.30%

Nevus-Scalp	38	3	7.90%

Nevus-Spitz	49	6	12.20%

**Subtotal**	**176**	**Percent Positive**	**9.10%**

			

**Melanoma**			

Superficial Spreading	71	57	80.30%

Spindle cell/desmo-plastic	3	2	66.70%

Nodular	45	42	93.30%

In Situ	8	6	75.00%

Metastatic	40	33	82.50%

**Subtotal**	**167**	**Percent Positive**	**83.80%**

**Total Cases**	**500**		

## Discussion

Presented is an analysis of 500 cases, comprised of 157 nevi, 167 dysplastic nevi, and 176 melanoma cases. The four-fluor FISH assay correctly identified 83.8% of melanomas, and 98.1% of all benign nevi.

The four-fluor FISH panel utilized in this study identified 83.8% of true melanomas. This is consistent with previous FISH and array comparative genomic hybridization studies, and supports the previous literature which has clearly determined that melanomas typically exhibit a grossly altered genetic complement. In terms of clinical utility, the four probe panel is an effective compromise between cost, technical complexity and assay sensitivity. A four probe panel represents the maximum number of FISH probes that can be co-hybridized to a single section. Increasing the number of probes would increase the sensitivity, but also increase the technical complexity of the assay and associated clinical cost.

Significantly, the RREB1 probe, at 60% exhibited the highest sensitivity for melanoma, while the CCND1 probe and MYB probes exhibited significantly lower sensitivities at 38% and 37% respectively. The derived cutoff values for each probe are presented in Table 3. Briefly, a case was considered positive if greater than 16% of scored cells for the RREB1 loci contained more than 2 signals; or greater than 22% of CCND1contained more than 2 signals; or more than 53% of nuclei contain more 6p25 than centromere 6 signals; or more than 42% of nuclei contain fewer 6q23 than centromere 6 signals. The cutoff values presented in this study were similar, although lower than the previously published values of (29%, 38%, 55%, and 42% respectively) [7]. These differences are most likely due to variation in scoring technique, cell selection, and equipment (manual verse automated) between the two studies. Applying the cutoff values of Gerami et. al. (2009) to the dataset of this study resulted in a sensitivity and specificity of 65% and 98.7% respectively (data not shown). These differences highlight the importance of performing site specific validations for sensitivity and specificity prior to clinical application.

**Table 3:** Derived Cutoff values by Probe

Loci	Cutoff Value
RREB1>2	16%

CCND1>2	19%

RREB1>cen6	53%

MYB< cen6	42%

Review of the benign lesions determined the assay identified genetic abnormalities in less than 1% of nevi without atypia (compound, junctional, congenital, and intradermal), while genetic abnormalities were identified in 6.3%, 6.7%, and 10.3% of nevus identified with mild, moderate and severe atypia respectively. These results are suggestive of a correlation between the severity of dysplasia and an increased probability of genetic abnormality and progression to melanoma. As it is standard practice to fully excise all dysplastic nevi, there is no opportunity to follow this cohort to provide direct evidence of morphologic progression to melanoma. While there is insufficient evidence to confirm the absolute malignancy of a lesion based solely on FISH analysis, the authors agree with the previous study's conclusions that the FISH positive dysplastic nevi group is of a significantly higher risk of progression to melanoma.

The assay did not identify one or more of the genetic abnormalities in 16.2% of all confirmed melanocytic lesions. This result infers that while these regions may be important in pathogenesis in some melanoma, they are clearly not causal in all melanomas. Both FISH and comparative genomic hybridization studies have previously been unable to identify a single genetic marker, or set of markers present in all samples, suggesting that the phenotypic representation of melanoma is actually comprised of several (or more) genetic "families", each with a unique underlying set of down regulated tumor suppressors and activated oncogenes driving pathogenesis. Similar degrees of genetic heterogeneity has been observed and studied in other cancers, and molecular characterization is being utilized clinically as an adjunct to traditional phenotypic markers to assist in diagnosis and treatment of many cancers including invasive breast carcinoma [3], lung cancer [17,18], prostate cancer [19], sarcomas [20], and renal tumors [21]. In invasive carcinoma of the breast, molecular profiling is routinely utilized in conjunction with traditional pathology to sub classify the tumors into molecular classes (basal-like, luminalA, luminal B, ERRBB2/Her2+), with different prognosis and different responses to specific therapies [3].

## Conclusions

These results are consistent to the current literature models for cancer progression, characterized by a defined initiating event, followed by a series of degenerative progressive genetic mutations, phenotypic de-differentiation, activation of tumor oncogenes, inhibition of tumor suppressor genes, until the cell reaches a state of de-differentiation where invasion and metastatic potential is possible. While the genetics behind this progression is well studied in several malignancies, there is still significant disagreement in the melanoma community as to the correlation (if any) between genetic instability and phenotypic dysplasia or atypia. While there continues to be significant discussion in pathology texts on the role of atypic presentation in the progression of melanocytic nevi to melanoma, genetically, it is clear that the process from melanocyte to melanoma requires multiple initiating events, followed by genomic instability, and finally progression to phenotypically evident malignancy.

Based on these results, the four-fluor FISH assay accurately identifies a molecular signature which is consistent with lesions that have invasive and metastatic potential. Lesions with these genetic qualities should be considered as possessing a genetic signature consistent with invasion and metastatic potential, even if this is not evident by morphology alone.

Clinically, the utility of this assay is predicated on the high specificity, and even in the absence of a conclusive melanoma diagnosis, the authors believe that a positive FISH case with corresponding atypical morphology should be treated as a high risk lesion and may warrant a wider excision, and more routine follow-up. Due to the moderate sensitivity of 83.8%, care must be taken in the analysis of dysplastic nevi which are negative by FISH, with the inference being that almost 17% of melanomas are driven by a molecular profile which is undetected through this assay. Conversely, a negative FISH result may provide additional re-assurance to the pathologist in cases where a benign result is expected, and excision is difficult or undesired. When accompanied by morphological risk factors, a positive FISH result is a valuable diagnostic tool for the identification of high risk lesions.

## Competing interests

Robert Gasparini (RG) and Mathew Moore (MWM) are both employees of NeoGenomics, a company specializing in clinical oncology, including the diagnosis of melanocytic lesions. This study was performed in part to determine if FISH could be used at NeoGenomics to improve the accuracy of diagnosis of challenging melanocytic lesions.

## Authors' contributions

MWM and RG conceived of the study, and participated in its design and coordination. MWM supervised and carried out the studies. MWM wrote the manuscript, and RG contributed to the manuscript review and draft. All authors read and approved the final manuscript.
